# An Unexpectedly Broad Thermal and Salinity-Tolerant Estuarine Methanogen Community

**DOI:** 10.3390/microorganisms8101467

**Published:** 2020-09-24

**Authors:** Lynsay I. Blake, Angela Sherry, Obioma K. Mejeha, Peter Leary, Henry Coombs, Wendy Stone, Ian M. Head, Neil D. Gray

**Affiliations:** 1 School of Natural and Environmental Sciences, Newcastle University, Newcastle upon Tyne NE1 7RU, UK; angela.sherry@northumbria.ac.uk (A.S.); obioma.mejeha@futo.edu.ng (O.K.M.); peter.leary@uzh.ch (P.L.); h.coombs@pageantmedia.com (H.C.); ian.head@newcastle.ac.uk (I.M.H.); 2 Department of Biosciences, Durham University, Lower Mount Joy, South Road, Durham DH1 3LE, UK; 3 Hub for Biotechnology in the Built Environment, Department of Applied Sciences, Northumbria University, Newcastle upon Tyne NE1 8ST, UK; 4 Department of Microbiology, Federal University of Technology, Owerri P.M.B. 1526, Nigeria; 5 Water Institute and Department of Microbiology, University of Stellenbosch, Stellenbosch 7602, South Africa; wstone@sun.ac.za

**Keywords:** methanogenesis, methanogen community function, methanogen community structure

## Abstract

Moderately thermophilic (T_max_, ~55 °C) methanogens are identified after extended enrichments from temperate, tropical and low-temperature environments. However, thermophilic methanogens with higher growth temperatures (T_opt_ ≥ 60 °C) are only reported from high-temperature environments. A microcosm-based approach was used to measure the rate of methane production and methanogen community structure over a range of temperatures and salinities in sediment from a temperate estuary. We report short-term incubations (<48 h) revealing methanogens with optimal activity reaching 70 °C in a temperate estuary sediment (in situ temperature 4–5 °C). While 30 °C enrichments amended with acetate, H_2_ or methanol selected for corresponding mesophilic trophic groups, at 60 °C, only hydrogenotrophs (genus *Methanothermobacter*) were observed. Since these methanogens are not known to be active under in situ temperatures, we conclude constant dispersal from high temperature habitats. The likely provenance of the thermophilic methanogens was studied by enrichments covering a range of temperatures and salinities. These enrichments indicated that the estuarine sediment hosted methanogens encompassing the global activity envelope of most cultured species. We suggest that estuaries are fascinating sink and source environments for microbial function study.

## 1. Introduction

Methane (CH_4_) is an important greenhouse gas, with a global warming potential over 100 years (GWP_100_) between 28 and 36 times that of carbon dioxide (CO_2_). It also contributes to global biogeochemical cycles [1,2] and is an important energy resource [3]. Methane is mainly produced biogenically [1], by methanogens, which provide a terminal process in the anaerobic degradation of organic matter.

In temperate, tropical and high-latitude environments at low in situ temperatures, methane production is generally dominated by mesophilic methanogens, carrying out acetate fermentation (acetoclastic methanogenesis). When acetoclastic methanogenesis is inhibited, or in situ temperatures are increased, carbon dioxide reduction coupled to hydrogen oxidation plays an increasingly important role (hydrogenotrophic methanogenesis) [4].

Although thermophilic methanogens are found in extreme environments such as hot undersea mud volcanoes, hydrothermal vent sediments, or deeply buried in the Earth’s sub-surface (T_opt_ ranging from 50 to 98 °C) [5], they are not ubiquitous [6,7,8,9]. Many enrichment studies of flooded soils, wetlands and sediments have shown that above in situ temperatures, distinct populations of moderately thermophilic methanogens (hydrogenotrophic methanogens: T_opt_ ~40–50 °C, T_max_ ~55 °C) become active after long lag phases (i.e., of weeks to months) [4,10,11,12,13,14,15,16], implying that these moderately thermophilic methanogens are minor inactive components of the in situ communities, potentially recruited by passive dispersal [17].

Estuaries play a unique interconnecting role between the terrestrial and marine environment [18]. They have been used to study the influence of environmental perturbation on in situ microbial community diversity, and function for specific microbial processes, including methanogenesis [19,20,21] and methanotrophy [18]. Interest is often primarily because, along with fjords and shallow coastal areas, estuaries are thought to contribute up to 75% of the marine global flux of methane to the atmosphere [22], despite making up only 16% of the total ocean surface area [23]. Therefore, in the future, estuaries may become more important methane source environments due to climatic and land use change enhancing nutrient and organic matter flow to them [24]. Many studies of methanogenesis indicate that the limits of methanogenic activity reach beyond those encountered under, or predicted for, in situ conditions [6,7,8,9,10,11,12,13]. Having previously identified aerobic methanotrophs with environmental tolerances for growth far beyond those encountered in situ in temperate estuarine sediments [18], we hypothesised that the methanogenic community may encompass a wide range of environmental tolerance (related to, but not necessarily limited by, environmental conditions and inputs from source environments). On this basis, we investigated the range and upper limits of methanogenesis in a temperate estuary. 

## 2. Materials and Methods

Sediment samples were taken from the Scotswood site, Tyne estuary, UK (54°58′47″ N 1°44′35″ W), using a 1 m length, 20 cm diameter perspex core. Membrane Inlet Mass Spectroscopy (MIMS) was used to qualitatively identify the in situ depth of the CH_4_ maximum. A quadrapole mass spectrometer (HAL3F-RC, HPR20, Hiden, Warrington, UK) was attached to an 8-way HPR40 inlet manifold, linked to 1 m stainless-steel gas inlet capillary tubes (Probe: 1.6 mm o.d, 0.5 mm i.d). The final centimetre of each probe was enclosed in a 100 μm thick silicone rubber sleeve (gas permeable membrane) overlying 4 rows of 1 mm diameter, pre-drilled holes. The gas permeability of this membrane enabled the (non-selective) diffusion of gases from the sample to the quadrapole mass spectrometer under a vacuum (created by a rotary vacuum pump, E2MI.5; Boc Edwards, UK). The mass to charge ratios of characteristic positive ions of the gases of interest (i.e., CH_4_ and Ar) were measured simultaneously and the output presented as a proportion of the total gas pressure (Torr) using the MA soft 5.8 software package (Hiden, Warrington, UK). The probe was positioned at the lowest part of the intact sediment core, a stable gas reading was taken and the probe was moved upwards by 5 cm. This process was repeated until the probe reached the upper region of the sediment, at this point 1–2 cm increments were made. For the simple purpose of identifying methane maxima, mass selective measurements (Torr) were corrected for matrix effects using conservative gas readings, i.e., Ar, by converting the raw gas readings into a ratio of CH_4_/Ar. The core was then sectioned under anoxic conditions (extruded into a glove box under a positive pressure of oxygen-free nitrogen). Sediment from the CH_4_ maximum (10–14 cm) was homogenised and refrigerated (4–5 °C) under anoxic conditions until microcosm set-up.

Triplicate microcosms (12 mL volume) were set up as in reference [6], with sulfate-free anaerobic enrichment medium (1 mL), homogenised sediment (0.5–1.0 g), and amended with methanogenic substrates, i.e., acetate (final concentration 10 mM), methanol (final concentration 10 mM), or H_2_/CO_2_ (4:1 in headspace 10 mL), alongside triplicate unamended controls. Multiple sets of these microcosms were set up and incubated for 50 days at temperatures of 5, 20, 30, 40, 50, 60 and 70 °C Additional control microcosms were treated with 2-bromoethane sulphonate (BES; 10 mM final concentration) and incubated at 30 °C. Additional sets of microcosms were similarly set up at 30 and 60 °C amended with NaCl medium (1, 15, 28, 46, 82, 114 and 137 NaCl g/L). Headspace methane was measured periodically using a Carlo ERBA HRGC 5160 GC-FID fitted with a Chrompak PLOT fused silica capillary column (30 m × 0.32 mm) using helium as a carrier gas. CH_4_ was quantified on the basis of peak area and calibrated using CH_4_ standards (Scientific and Technical Gases Ltd., Newcastle-under-Lyme, UK). Please note that methane production in the BES (2-bromoethane sulphonate; 10 mM final concentration) controls was not measurable. After 50 days, triplicate sets of microcosms were sacrificed for microbial community analysis. Rates of methanogenesis were calculated from the linear accumulation of methane per gram of sediment dry mass (DM). For the temperature experiment rates were calculated from the initial 7 day period of incubation. and for the salinity experiment were based on the maximum rate of accumulation. Rates of amended and unamended microcosms were compared statistically by one-way analysis of variance (ANOVA; Minitab 17, Minitab Ltd., Coventry, UK).

Triplicate microcosms sacrificed for molecular analysis were stored at −20 °C prior to DNA extraction. Extractions were carried out on each of the triplicate microcosm slurries (~0.25 mL) using a FastPrep Ribolyser (Hybaid Ltd., Hampshire, UK) and a BIO 101 FastDNA Spin Kit (for soil; Q-BioGene, Cambridge, UK), according to the manufacturer’s instructions. DNA was quantified using a Qubit^®^ dsDNA HS Assay Kit (Life Technologies, Carlsbad, CA, USA). The procedure stated in reference [25] was followed for microbial community structure analysis. Specifically, primer set F515/R926 [26] was used to PCR amplify the variable V4/V5 region of the archaeal and bacterial 16S rRNA gene. Primer F515 in addition to the target sequence also contained a PGM™ (Personal Genome Machine) linker primer/adapter, a Golay barcode (unique for each sample amplification) and a barcode spacer. R926 contained a truncated P1 (TrP1) adapter at the 5′ end [27]. Reactions were carried out using Bioline’s TaqMan DNA amplification kit (Bioline, UK) with conditions of 95 °C for 3 min followed by 30 cycles (1 min at 95 °C, 45 s at 55 °C, 1 min at 72 °C) and finally, 10 min at 72 °C. Amplicons were purified using an Agencourt Ampure XP purification Kit (Beckman Coulter Ltd., High Wycombe, UK) and quantified using a Qubit^®^ dsDNA HS Assay Kit (Life Technologies, USA). Amplicons were then pooled into an equimolar library of 500 pM DNA. Sequencing templates were generated by attaching the DNA samples to ion sphere particles using the Ion PGM™ Template OT2 400 kit (Life Technologies, USA), based on the manufacturer’s instruction, and using the Ion OneTouch™ 2 Instrument and the Ion OneTouch™ ES (Enrichment System). Sequencing was performed using the PGM™ sequencing Platform with the Ion PGM™ sequencing 400 kit followed by filtering to remove low-quality and polyclonal sequences. Data was analysed by the Qiime2 (Quantitative Insights into Microbial Ecology 2) pipeline (https://qiime2.org/) [28] to trim and cluster sequences into amplicon sequence variants (ASVs), assign taxonomies and generate representative sequence and ASV frequency outputs. Sequences have been deposited in the NCBI’s Sequence Read Archive (SRA) available under Bio Project PRJNA577052. Statistical Analysis of taxonoMic and functional Profiles (STAMP v2) [29] was used to compare taxonomic profiles between treatment groups and carry out Principle Component Analysis (PCA). Phylogenetic distance analysis was conducted using the Jukes–Cantor correction for multiple substitutions at a single site and the neighbour joining method using MEGA version 7.0 [30].

## 3. Results and Discussion

Methane production was measured in both low- (5–30 °C) and high (40–70 °C)-temperature River Tyne incubations after 48 h. In this time period, methane accumulation in unamended microcosms was low (e.g., reaching 0.74 ± 0.15 μmol CH_4_ g sediment (DM) at 20 °C, and 0.01 ± 0.005 μmol CH_4_ g sediment (DM) at 60 °C). Addition of methanogenic substrates resulted in increased methane accumulation (compared to unamended microcosms) at both these temperatures. This increase was particularly pronounced in H_2_/CO_2_ amended microcosms incubated at high temperatures (where in the first 48 h, methane accumulated to 32.38 ± 13.29 μmol CH_4_ g sediment (DM) at 60 °C). This response is in contrast with previous studies [10,11,12,13,14,15,16,31,32] where incubation of sediment/soil for an extended period of time, i.e., weeks to months, was required for the measurement of high-temperature methane production.

**Figure 1:**
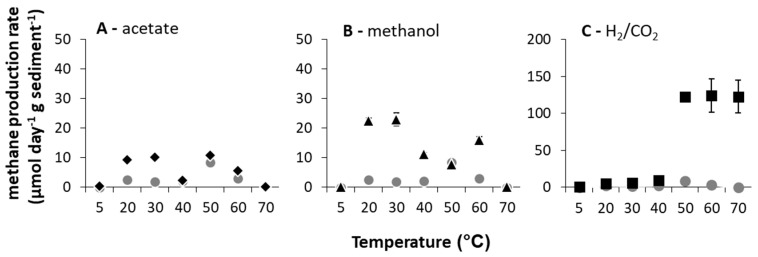
Methane production rates calculated between days 2 and 7 of incubation (μmol CH_4_ g^−1^ d^−1^ sediment (DM) ± SE (standard error), *n* = 3) over the incubation temperature range of 5 to 70 °C with methanogenic substrate addition ((**A**) acetate = black diamonds, (**B**) methanol = black triangles, (**C**) H_2_/CO_2_ = black squares), and without substrate amendment = grey circles).

When methane production rates were calculated (days 2–7), clear broad (potentially bimodal) optima (T_opt_) at these low- (5–40 °C for acetate and methanol amended microcosms and 30–40 °C for H_2_/CO_2_ amended microcosms) and high (50–70 °C for acetate and methanol amended microcosms and 50–70 °C for H_2_/CO_2_ amended microcosms)-temperature ranges existed (Figure 1). At high temperatures (≥50 °C), methanogenesis rates were greatest with H_2_/CO_2_ relative to unamended controls (pairwise comparisons, *p* < 0.05 at 50–70 °C).

This response is partially consistent with studies of lake sediment [4,10,16] and rice paddy field samples [11,13,32,33,34] incubated at elevated temperatures (i.e., ≤50 °C). In such studies, hydrogenotrophic methanogenesis was most enhanced in incubations above 40 °C and often found exclusively responsible for methane production [34], consistent with predictions for the influence of temperature [35]. In the River Tyne, though, the upper limit of methanogenesis was above 70 °C, the T_opt_ spanned 50–70 °C and the maximum rates of methanogenesis were the highest reported from any temperate enrichment (i.e., 121 ± 1.88 μmol CH_4_ g^−1^ sediment (DM) d^−1^ at 50 °C and 123 ± 22.32 μmol CH_4_ g^−1^ sediment (DM) d^−1^ at 70 °C when amended with H_2_/CO_2_). In fact, only one other study of methane production from a low-temperature natural environment (Lake Baldegger, in situ temperature 4–5 °C) has reported any methane production at 70 °C [16], after 30 days of incubation.

**Figure 2:**
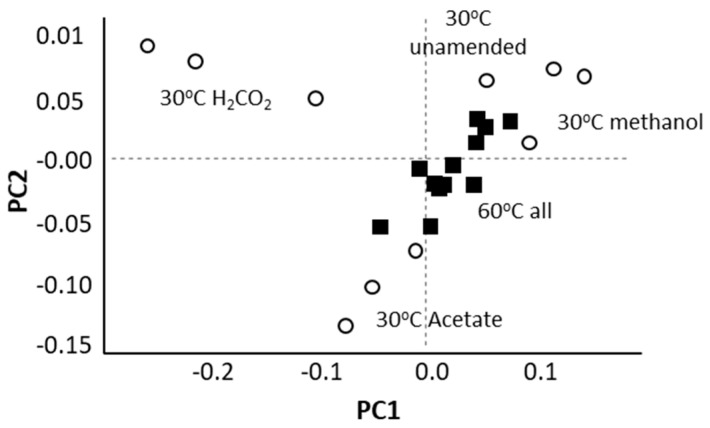
A Principal Component Analysis (PCA) based on an analysis of archaeal ASV (amplicon sequence variants) frequencies within 16S rRNA sequence libraries constructed from methanogenic enrichments indicates that archaeal communities were distinct based on substrate amendment at 30 °C (white circles) but not at 60 °C (black squares).

At 30 °C, different substrates ammendmentsselected for the different methanogenic archaeal communities. At 60 °C, this was not the case (Figure 2).

**Figure 3:**
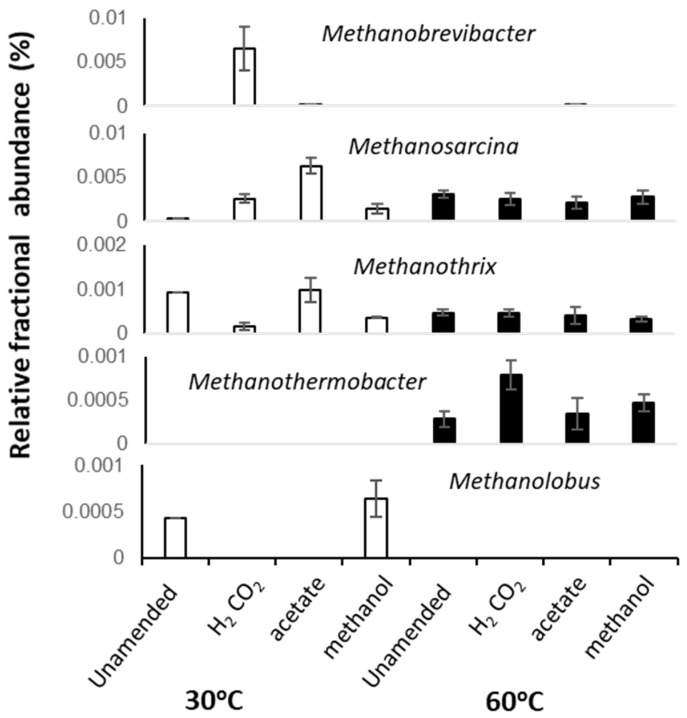
Average fractional abundances of selected dominant archaeal taxa identified in 16S rRNA sequence libraries from replicated 30 and 60 °C methanogenic enrichment microcosm. Error bars represent 1 × SE (*n* = 3).

**Figure 4:**
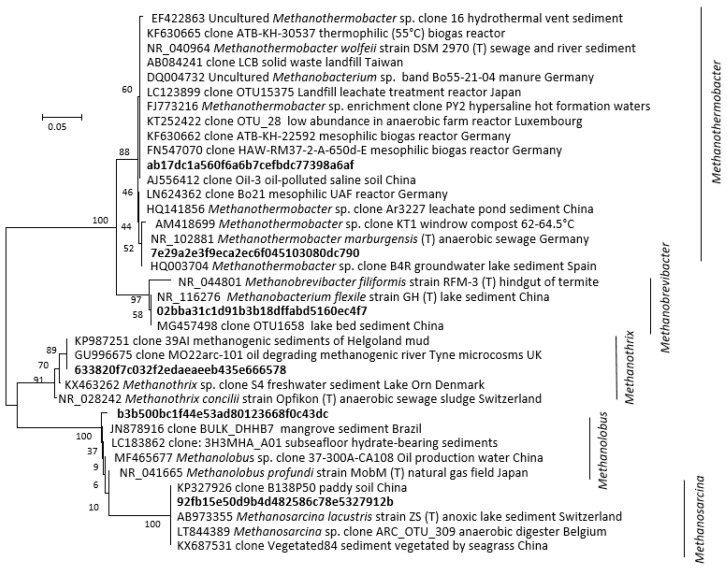
Phylogenetic distance tree (Neighbour-Joining) of key archaeal 16S rRNA amplicon sequence variants (ASVs) identified in the methanogenic microcosm and their close relatives. The tree was inferred from 248 positions. Individual codes for ASVs were assigned during pipeline analysis. The percentage of replicate trees in which the associated taxa clustered together in bootstrap analysis (1000 replicates) are shown next to the branches.

At 30 °C, methanol and H_2_/CO_2_ enriched for the methylotrophic (*Methanolobus*) and hydrogenotrophic (*Methanobrevibacter*) genera respectively (Figure 3 and Figure 4), and acetate enriched *Methanosarcina*. Consistent with the metabolic flexibility of this genus, *Methanosarcina* sequences were recovered from all incubations irrespective of substrate amendment (Figure 3). In contrast, hydrogenotrophic *Methanothermobacter* [36] were enriched in all 60 °C microcosms but were absent at 30 °C (Figure 3 and Figure 4). However, *Methanothermobacter* must have been present and viable initially in appreciable numbers to explain the short lag phases observed prior to detection of methanogenesis (<48 h in all high-temperature microcosms). Close relatives of the *Methanothermobacter*, not surprisingly, have been identified in, or have been isolated from, high-temperature environments (temperature range 50–70 °C), including sewage sludge, thermophilic anaerobic digesters, high-temperature oil field production waters (Figure 4), hot springs and solfatara [37]. However, close matches were also found with sequences recovered from moderately low-temperature systems (temperature range 5–40 °C), including lake sediments, temperate and tropical soils and sediments, mesophilic anaerobic digesters, cattle manures, composts and sediment from an artificial lake (Figure 4). Critically, studies of cold environments had not explicitly targeted or mentioned these thermophilic methanogens or their activities.

**Figure 5:**
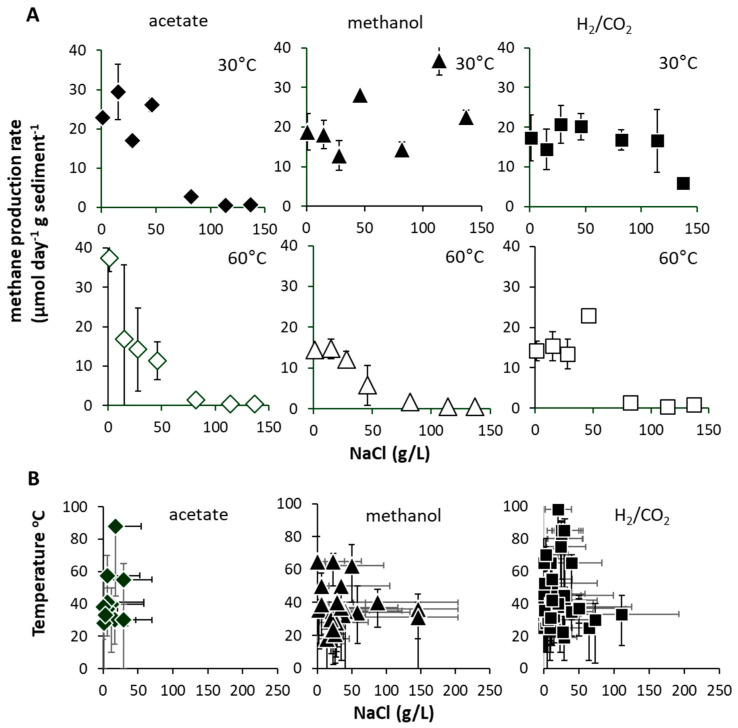
(**A**) Maximum methane production rates (μmol CH_4_ g^−1^ d^−1^ sediment (DM) ± SE, *n* = 3) at 30 and 60 °C (with substrate addition, acetate = white diamonds, methanol = white triangles, H_2_/CO_2_ = white squares) when incubated with 1 to 137 g/L NaCl. (**B**) Summary of global cultured methanogen temperature vs. NaCl optimum and range (indicated by acetate = black diamonds, methanol = black triangles, H_2_/CO_2_ = black squares). Data in (**B**) sourced from reference [5].

It has been proposed that the viability of such ‘extreme thermophilic species’ in low-temperature environments may result from low minimum growth temperatures (22 °C) [38]. Certainly, a *Methanothermobacter* sp. that dominated an enrichment culture from a high-temperature oil field water production [39] (Figure 4) after transport and 4 °C storage for months demonstrated that at least some remain viable following inactivity at low temperatures. Regardless, the short lag phases observed in the current study (<48 h) suggest constant replenishment of *Methanothermobacter* into the Tyne estuary sediment, from terrestrial or marine sources, e.g., landfill, hot geological, industrial, or agricultural habitats [40]. Given the likely passive dispersal from these sources [41,42], we attempted to resolve their mesophilic and thermophilic origins by determining salinity tolerances as a secondary niche dimension (Figure 5A). These tolerances were then compared with those of cultured methanogens for which both temperature and salinity ranges have been reported (Figure 5B) [5].

We found no strong evidence for low-salinity source environments, and surprisingly, found that the Tyne estuary methanogens actually exhibited tolerances and optima similar to those determined collectively for all cultured methanogens. Specifically, 30 °C mesophilic hydrogenotrophic and methylotrophic methanogens showed high-salinity tolerances (≤137 gL NaCl), but acetoclastic methanogens did not (≤50 gL NaCl, Figure 5A) [43]. In contrast, 60 °C Thermophilic hydrogenotrophic, methylotrophic and acetoclastic methanogens were all limited to <50 gL NaCl. This pattern aligns with the global inventory of thermophilic methanogens (T_opt_ > 40 °C), which generally show moderate salinity tolerances (e.g., average NaCl_opt_ 14.6 gL, average NaCl_max_ 45 gL) (Figure 5B) [5], and suggests that the Tyne estuary mesophilic and thermophilic methanogen communities encompass close to the full global range of salinity tolerance for this group of organisms.

In the face of global challenges such as climate change, energy production and food security, there are increasing calls for ”coordinated, cross-disciplinary efforts to understand, predict and harness microbiome function” [44]. However, harnessing the potential of the global microbiome requires understanding of the range of function the microbiome can offer and the most interesting and useful sites for investigation. Exploration of extreme environments has been pivotal in uncovering microorganisms with exploitable function, but fewer studies have focused on understanding the full microbial potential of more accessible environments. This enrichment study, when considered alongside a recent meta-analysis of DNA-based global estuarine archaeal biodiversity studies [45], builds a picture of estuarine sediments holding unexpectedly broad, demonstrably viable archaeal function, and unexpectedly rich archaeal biodiversity (spanning the rare to the ubiquitous) [45]. Estuaries, therefore, are potentially useful microbial sink and source environments of microbial function.
